# Objective and bias-free measures of candidate motivation during job applications

**DOI:** 10.1038/s41598-021-00659-y

**Published:** 2021-11-09

**Authors:** Mitchel Kappen, Marnix Naber

**Affiliations:** 1 Experimental Psychology, Helmholtz Institute, Faculty of Social and Behavioral Sciences, Utrecht University, Utrecht, The Netherlands; 2 Department of Head and Skin, Ghent University, University Hospital Ghent (UZ Ghent), Ghent, Belgium

**Keywords:** Psychology, Human behaviour

## Abstract

Society suffers from biases and discrimination, a longstanding dilemma that stems from ungrounded, subjective judgments. Especially unequal opportunities in labor remain a persistent challenge, despite the recent inauguration of top-down diplomatic measures. Here we propose a solution by using an objective approach to the measurement of nonverbal behaviors of job candidates that trained for a job assessment. First, we implemented and developed artificial intelligence, computer vision, and unbiased machine learning software to automatically detect facial muscle activity and emotional expressions to predict the candidates’ self-reported motivation levels. The motivation judgments by our model outperformed recruiters’ unreliable, invalid, and sometimes biased judgments. These findings mark the necessity and usefulness of novel, bias-free, and scientific approaches to candidate and employee screening and selection procedures in recruitment and human resources.

## Introduction

The subjective judgment of employees and candidates on organization- and job-fit during recruitment and human resources selection procedures can be unfair. Numerous investigations have revealed the often defective and biased nature of subjective evaluations of behavioral and personal aspects in interviews^1^. In short, professionals hardly agree on their judgments (low interrater reliability) and judgments are based on and influenced by irrelevant factors (low validity) during unstructured interviews. While structured interviews and the training of human resource personnel may solve these issues to some degree, interviews both with and without a trained eye characterize the major approach to hiring and yield substantially lower interrater reliability levels than commonly accepted^1^. As such, the lucky candidates are the white, attractive, young males: they are most likely to get hired or promoted^2–4^. The unlucky job applicants go back home empty-handed, may it because of their physical appearance, the interviewer’s pessimistic mood^5,6^, or the interviewer’s limited and inaccurate behavioral observations^7^ that play a significant though unfortunate role in unsubstantiated decision-making in human resources^8^. Despite these facts, we hypothesize that the core of this problem originates from human’s tendency to underestimate how prone they are to error in inferring people’s intentions, skills, and mental states during interaction, and how deceptively confident they are in relying on their subjective judgments. But how can we deal with such human nature that so adversely continues to stall diversity and inclusivity in today’s society? Although the field is still in its infancy, we will here show that artificial intelligence and computer modelling can play a crucial role in solving the absolute necessity for more objective screening procedures^9^.

Recent advancements in artificial intelligence, big data, and modelling replace human raters by objectively evaluating candidates through text mining their accomplishment records^10–14^. When these types of models are properly trained, they produce no biases. Another relevant development is the production of language-based personality assessments during video interviews but the models have so far resulted in inconclusive findings^15^. An alternative and perhaps more successful approach could be the utilization of computer vision techniques to objectively measure facial behavior to unveil how these relate to cognitive processes^16^, mental wellbeing^17^, and personality (e.g.^18–21^). Many of these traits are relevant predictors of job performance and satisfaction^22,23^. When essentially trained on unbiased datasets, computer vision and machine learning models may provide reliable and consistent ratings of relevant behavior.

Here we make the first but modest step to generate more objective behavioral video assessments in recruitment and human resources procedures by training machine learning models on introspective judgments of candidates. We have used automated analyses of facial action units to predict the motivation of candidates in a simulated job selection experience. In this exploratory study, we chose to predict motivation because it is one of the strongest predictors in job success^22,23^, job satisfaction^24^, and job retention^25^. Different from these previous studies that investigated the importance of motivation to go to work and perform, we focus however, on a slightly different form of motivation, namely a candidate’s motivation to start working for a company. To the best of our knowledge, this construct has received no attention from the literature. We propose that the measurement of a person’s motivation to work for a company in a prescreening assessment, before an actual (un)structured interview takes place. It may also tackle the financial burden brought about by standard, time-consuming application procedures, and may potentially prevent job-hopping, and unequal, inefficient hiring^26^.

The current study presents evidence that, in an application training context, (i) recruiters disagree on how motivated applicants are, (ii) recruiters disagree with candidates themselves on how motivated they are, (iii) a machine learning model successfully dissociates the most motivated from the least motivated candidates by using action unit activity and emotional expressions as predictors, and (iv) recruiters correctly pay attention but incorrectly weigh relevant facial markers to determine a candidate’s motivation levels.

## Results

**Figure 1:**
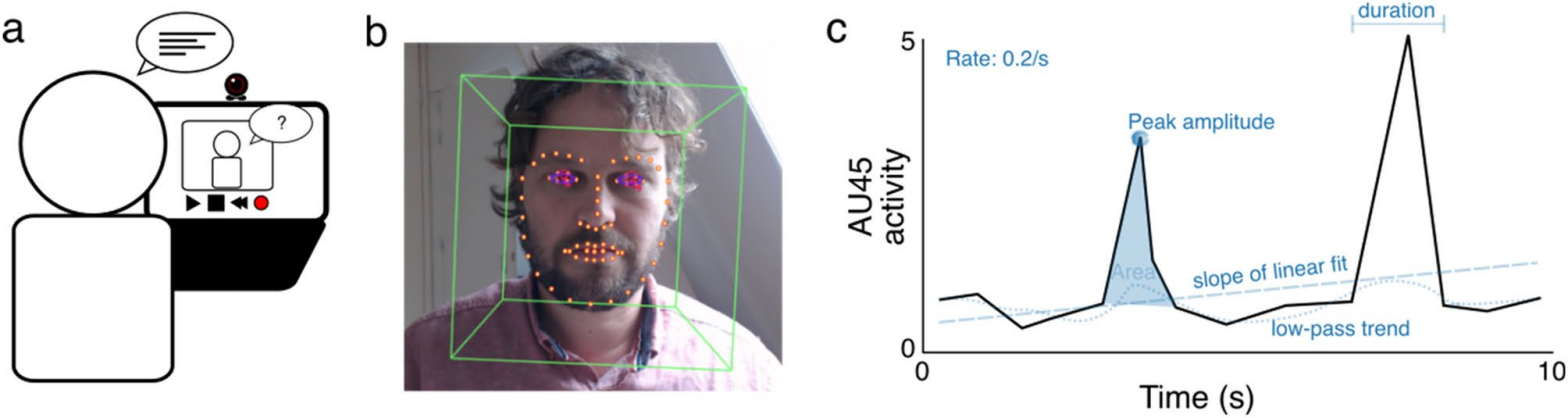
Procedure and feature extraction. (**a**) Candidates’ (participants) faces were recorded while they conducted an automated interview on a computer. (**b**) Example of detected facial markers (orange circles) for action unit activity measurements (person in the image is one of the authors, not a participant, and has approved the use of this image). (**c**) Schematic example of a recording of action unit 45 (eye blinks). The behavioral features extracted from the signal are highlighted in blue.

A total of 154 students participated in this study with the intention to practice an online assessment in a simulated application setting in the role of a candidate for a job position. We made the candidates well aware that no actual job position was vacant. We also chose a notorious Dutch-British oil company as a mock employer to ensure that candidates varied substantially in their degree of feeling affiliated with the company. The candidates sat behind a computer and took part in an online, automated interview while their faces were recorded with a webcam. They watched three separate pre-recorded videos, each in which a recruiter asks them a different question. Candidates responded verbally after each question (Fig. 1a). Computer vision software detected the activation of facial action units (AU) and basic emotional expressions during the entire structured interview (see “Methods”, for details). This software uses neural networks to detect faces, relevant 2D facial coordinates (see orange dots in Fig. 1b), and geometrical transformations to compute 3D Euclidian distances between markers as AU activity per video frame (see Supplementary Table 1, for AU and emotion labels). We then broke down the activity of each AU and emotional expression into measures indicative of several time and strength-dependent AU dynamics to be used as input to an objective model (Fig. 1c). Candidate motivation was measured on a 10-point Likert scale and determined by four different parties: (1) by the candidates themselves after the interview; (2) by a candidate-based motivation model (CBMM), trained on the scores of the candidates (model 1); (3) by several recruiters that watched a subset of the candidate videos; and (4) by a recruiter-based motivation model (RBMM), trained on the scores of the recruiters (model 2).

The candidates’ introspectively determined motivation ratings varied substantially, that is across the full range of scores with a mean of 4.6 and a standard deviation of 2.5 (see Supplementary Figure S1a, for histograms). This indicates that a substantial number of candidates answered to be not motivated at all. The CBMM produced a similar distribution of motivation ratings (M = 4.8, SD = 2.2), while the recruiters’ scores showed a narrower distribution (SD = 1.3) elevated around a mean of 6.8.

**Figure 2:**
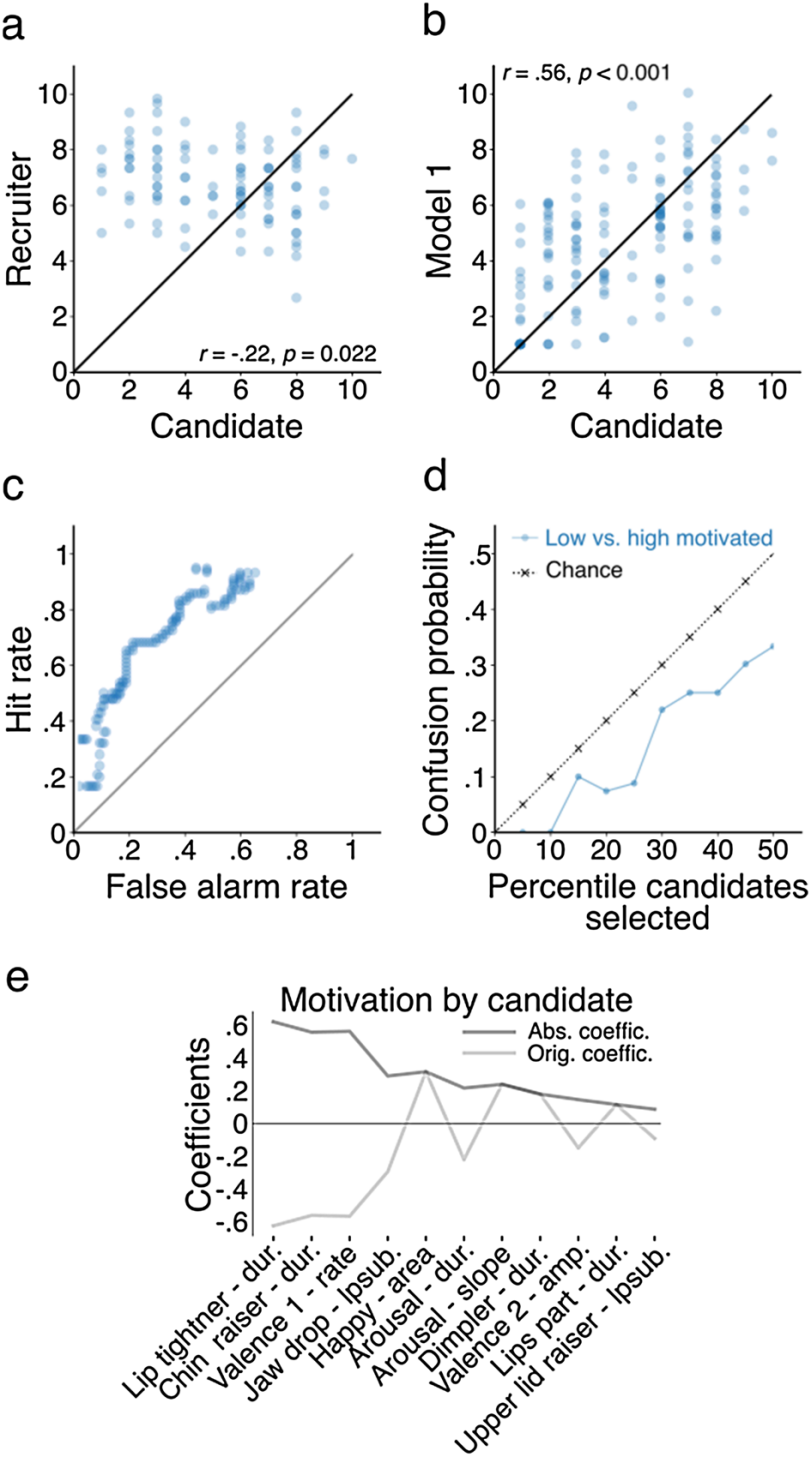
Predicting candidate evaluations. (**a**) Scatter plot correlation of motivation scores by candidates themselves (ground truth) versus recruiters. (**b**) Same as panel (**a**) but now for candidate versus CBMM model ratings. (**c**) Signal detection ROC curve with hit rate (sensitivity) as a function of false alarm rate (specificity). (**d**) Probability to confuse a CBMM-based high motivated candidate with a low motivated candidate. (**e**) Coefficients of features of the CBMM that determines scores of candidates themselves (dark grey line shows absolute values of the actual coefficients depicted in light gray).

Next, we scrutinized how well recruiters and the CBMM could determine the self-reported motivation levels of candidates. We observed a negative correlation between motivation ratings of recruiters and candidates themselves (Fig. 2a), meaning that recruiters rated more motivated candidates as less motivated and, vice versa, less motivated candidates as more motivated. One recruiter expressed a gender bias as she rated men as significantly more motivated than women (Women: M = 4.6; men: M = 5.9, *t*(102) = 2.48, *p* = 0.017; Other recruiters: *p* > 0.298; No age bias was found). Lastly, the recruiter group did not agree on the motivation levels of candidates as interrater reliabilities scored poor (Krippendorff’s *α* = 0.29). In sum, recruiters’ motivation judgments turned out to be unreliable, invalid, and sometimes biased.

The CBMM’s motivation ratings, however, correlated positively with the self-report ratings of candidates (Fig. 2b), indicating that action units and emotions were predictive of a person’s internal level of motivation. The model’s ratings contained no biases (e.g., women were rated as motivated as men; women: M = 4.8; men: M = 4.7, *t*(152) = 0.11, *p* = 0.911). To further investigate how well the model dissociated more motivated from less motivated candidates, we used signal detection theory to compute hit (i.e., correctly labelled candidate as more motivated) and false alarm (i.e., incorrectly labelled as more motivated) rates across the full range of possible motivation thresholds, each set to divide the more motivated from the less motivated, to generate an ROC-curve (Fig. 2c). When, for example, a recruiter’s goal was to select the top 25% most motivated participants (motivation score > 6.3), approximately half of the motivated candidates and only 10% of the less motivated candidates would be selected. When comparing random (chance) selection to CBMM selection as a function of percentile selected candidates (Fig. 2d)—selecting randomly would still produce better results than having recruiters subjectively select motivated candidates—the probabilities to confuse a more motivated candidate (e.g., > 75th percentile) for a less motivated candidate (e.g., < 25th percentile) was always lower for the model (e.g., 15% lower).

To better understand what the model used to determine motivation, we inspected the modelled weights (beta coefficients) per AU and emotion (Fig. 2e). The model’s feature weights indicated that relatively frequent positive expressions (valence) and long-lasting episodes of a tightened lip and raised chin predict *low* motivation levels in candidates. When taking all relevant features into account, we can conclude that the most motivated candidates showed small amounts of lip and jaw muscle activity but stronger expressions of positive emotions.

**Figure 3:**
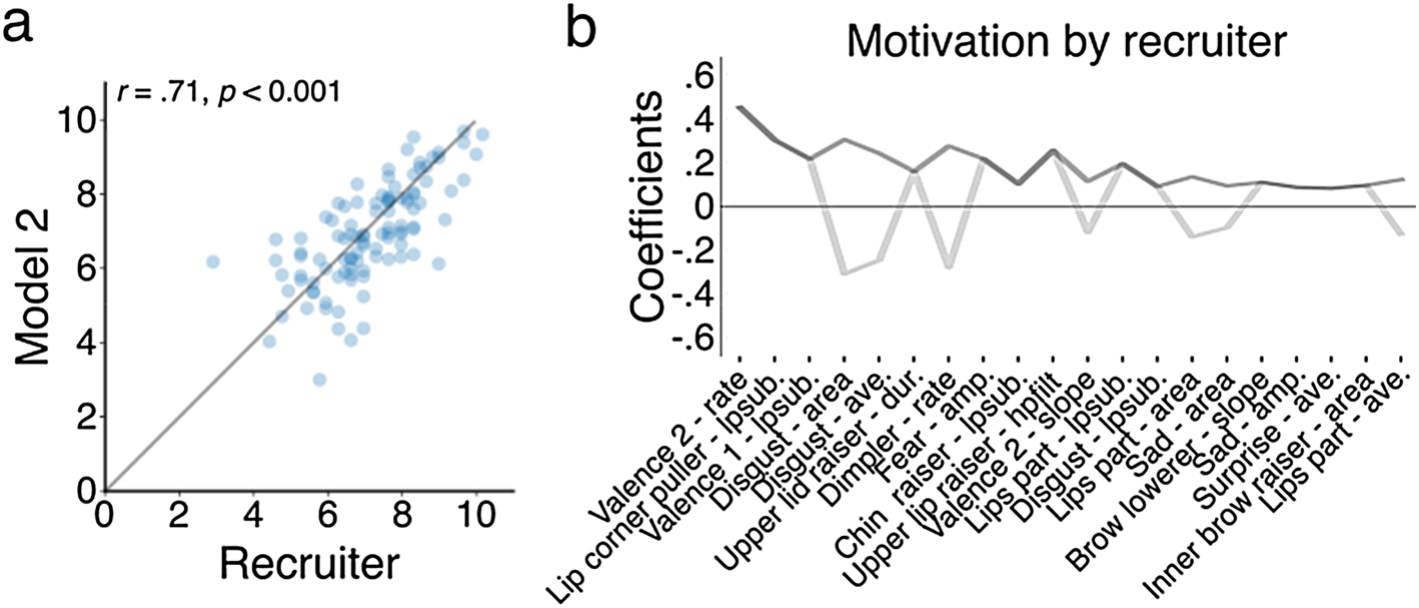
Predicting recruiter judgments. (**a**) Scatter plot correlation of motivation scores by recruiters (raters) versus CBMM. (**b**) Same as panel **e** in Fig. 2, but now for RBMM.

The recruiters either paid attention to different facial features or weighted them differently than the model, as confirmed by the lacking correlation between recruiter and CBMM model ratings (*r*(103) = − 0.10, *p* = 0.328). What type of facial change was it that recruiters then wrongly interpreted as a sign of motivation? To investigate this, we created a separate model (model 2; RBMM) that predicted the recruiters’ ratings. This model performed quite well (Fig. 3a) and coefficients indicated that frequent and relatively positive expressions, pulled lip corners (upward movement of mouth corners), and weak disgust expressions proved to be most relevant to recruiters to determine motivation (Fig. 3b). Remarkably, some of the AUs and emotions were relevant for both CBMM and RBMM. However, comparing the coefficients between the models for these predictors showed that the majority got weighted with opposite signs. This means that, while rating candidate motivation, recruiters paid attention to most AUs and emotions that a self-reported, highly motivated candidate would express, but assigned the wrong estimates to these features.

## Discussion

To summarize the results, recruiters’ observation-based ratings of motivation levels did not correspond to the candidates’ introspective suggestions. Recruiters’ judgments of nonverbal, video-recorded behavior are also unreliable. However, a computer model’s motivation ratings, trained on candidate’s self-reported ratings and based on measurements from candidate’s facial AUs and emotions extracted by artificial intelligence and computer vision algorithms, are reliable—a characteristic inherent to objective machine learning models—valid and unbiased. Post-hoc analyses indicated that recruiters expect, or perhaps unconsciously infer, that a high rate (not the intensity) of smiles and expressions with a positive valence reflect a state of motivation, while the occurrence of disgusted expressions, dimpled cheeks, and opening of the mouth reflects a state of *de*motivation. The importance of positive valence and rate of smiles for an interviewer’s favorability of candidates is in line with previous research^27–29^. Assuming that introspection approaches ground truth more closely than observation (see below for a more elaborate discussion), the CBMM suggests that recruiters may improve their judgments, however, by paying attention to the strength rather than the rate of happy expressions. They could also look for signs of *de*motivation by paying attention to long episodes of the tightening of the lips and the raising of the chin, and too frequent positive expressions. After inspection of the CBMM’s valuation of features, it is tempting to suggest that motivated candidates appear more serious and concentrated overall, and when expressing a positive emotion, it is expressed intensely.

This study for the first time demonstrates that introspection-based and observation-based motivation levels of candidates can be determined objectively with computer vision techniques and machine learning models. Previous studies examined only still images, observation-based behavioral ratings, or stable personality traits (for reviews, see^30–33^). Here we took this type of research a significant step forward by focusing on dynamic facial behavior recorded with a webcam, the prediction of unbiased and honest self-reports rather than of judgments by others based on subjective observations, and (temporary, task-evoked) state rather than (stable) trait behavior.

Considering the relatively small sample size of recruiters used in the current study—the main scope of this study is the prediction of self-reported motivation rather than judgments by recruiters—the more remarkable it is that we found a gender bias in one of the recruiter’s judgments, a well-known problem in personnel selection (e.g.^2^). Another relevant observation is the discrepancy in the range and overall mean of motivation scores between the recruiters and participants. The lacking overlap in score ranges may be easily solved by proper rater training, but this is not always performed in HR departments^34^ and, more importantly, it won’t take away the problematic negative correlation between the motivation scores of recruiters and participants. Instead, the here reported findings and computer vision model could help to train recruiters to better detect motivation levels.

One may question to what degree the self-reports of motivation serve as a reliable ground truth. It makes sense to assume that one has better access to his/her motivational state than others. Also, the simulated job application assessment and the notorious company that offered the mock vacancy prevented candidates to provide socially desirable answers. Indeed, the broad range of candidates’ motivation scores, with approximately half of the candidates indicating a demotivated state during the assessment, indicates that these self-reports are honest and reliable.

It should be noted that no standardized questionnaire yet currently exists to measure one’s work motivation in a prospective manner. Work motivation is often measured in active employees and is closely related to job satisfaction, success, and retention^22–25^. However, test-motivation (during job applications) has also been shown to be of a predictive outcome on job performance (and work-related motivation), presumably because of the focus on “will do” rather than “can do”^35,36^. This hints at the possibility that work motivation is a generic factor—a rigid mental state, not prone to change—a proposition that asks for psychometric validation in future research. Nonetheless, the psychometric quality of our motivation assessment is limited and future studies should consider the use of an organizational attractiveness scale^37^ or other validated questionnaires alike. Such scales are known to reflect a candidate's real intention to apply for the job^38^. Another interesting topic to pursue in future studies is the measurement of the occurrence of faking and posed emotions^33^ as well as the use of deep learning to gain more accurate predictions of the intensity of these emotions^39^. The participants that are not motivated but still aim to perform well during the interview, may also produce fake expressions more often and the question remains to what degree an objective model would be sensitive to faking. A multimodal approach that integrates phonetic and semantic properties of speech with computer vision analyses to determine sentiments may yield more accurate and representative results^40^. The same may be achieved with the implementation of automated emotion categorization algorithms (for a review see^41^) rather than the use of predefined emotions.

The measurement of a candidate’s motivation to work for a company is just the beginning and the here applied analysis procedure generalizes easily to the assessment of other selection-relevant aspects that enhance person-organization and person-job fit (e.g., stress). Although out of the scope of the current study, model performance can be improved, for example by training nonlinear models such as neural networks—though at the expense of interpretability—and by extending the measurement set with eye movements, head posture, and cardiac biomarkers through remote photoplethysmography^42^. Furthermore, to enhance accuracy, both top-down and bottom-up approaches should be used, leveraging symbolic as well as subsymbolic methods^43,44^.

Our results concur with previous demonstrations in other domains showing that artificial intelligence and computer models outperform humans on a variety of tasks^45^. We attribute the poor judgments of recruiters to three factors: first, attentional bottlenecks limit human’s ability to pay attention to multiple features in parallel^46,47^; Second, humans lack the skill to infer information from subtle facial changes unless they receive explicit feedback about the validity of markers^48^; Third, recruiters are not trained well as it is unlikely that they have ever received valid feedback from candidates (e.g., “I was not motivated at all to apply for this job”) because candidates provide socially desirable answers to be selected for a position. Despite these shortcomings, experts remain to overestimate their judgment skills, even when judgments and decision-makings negatively affect the wellbeing of others, like in medicine^49^. Although criticized heavily due to the lack of transparency about the underlying algorithms, several companies have recently begun to apply AI-based pre-screening of candidates^50^. Adopting AI in decision-making will nevertheless be a challenge for recruitment, human resources^9^, and other domains in the near future. This is due to feelings of human uniqueness, lack of control, and perception of threat when dealing with AI systems^51^.

## Conclusions

The current findings show the feasibility of computer vision models to deliver information on behavior and mental states. Reliable and accurate information about candidates will help humans make better decisions, potentially leading to impact systematic judgment issues at the root of discrimination and bias and more diversity and inclusivity. Educating people about these issues helps but a perhaps more efficient approach would be to let people experience at work that AI-selected colleagues perform just as well or better. People spend the majority of their time in teams and organizations thrive from motivated colleagues^23^. Propelling subjective assessments in recruitment and human resources into an objective assessment will thus be an effective program to ban biases across the classes of society in the long term.

## Methods

### Participants (candidates)

This study was conducted on 154 participants (age: M = 22, SD = 2, range = 18–26; 51 men, 53 women). Participants were recruited at the campus of a large European research university through flyers, social media, and word of mouth. Lacking fluency in Dutch was the only exclusion criterion. Participants provided written informed consent prior to the start of the experiment. The study was approved by and performed in accordance with all the relevant guidelines and regulations of the Faculty Ethics Assessment Committee of Utrecht University’s Faculty of Social and Behavioural Sciences under number 19-079. Information in the current manuscript that appears to lead to the identification of participants are not participants, but authors that have approved the use of this material (e.g. Fig. 1b). Participants either received financial compensation or study credits.

### Procedure

This research focused on the results of an assignment of a larger experiment in which participants (candidates) took part in training for job assessments. The participants partook in an automated, computerized interview, but also conducted a cognitive capacity test and filled in several personality questionnaires, but the analysis of these latter two parts is out of the scope of the current study.

Participants were invited to partake in a practice assessment as part of a mock job application process for a traineeship at a large Dutch-British oil company. Participants were aware of the fact that no real job opening existed but they were told to take the application seriously and act as if they were actually applying for a traineeship. As additional incentives to ensure participants behaved seriously, we rewarded participants with a monetary incentive (6 Euro) and presented the experiment as an opportunity to practice an online webcam assessment, which is rising in popularity as a prescreening tool^52^. A company with a notorious reputation was chosen to ensure that participants would vary substantially in their opinions about and thus their motivation to work for the company. They were further informed on what a traineeship generally entails, and what the contents of this traineeship could look like. The interview consisted of watching a video of a recruiter asking the participant a question, to which the participants then had to verbally respond. A question–answer block was conducted three times, each with a different question that is typically asked during interviews: (1) What motivates you to work for [company name], (2) Give an example of a goal you achieved and how you achieved this goal, and (3) Describe a moment in which you did not agree with fellow group members, and how you went about this. After the interview, all participants answered the question “To what degree are you motivated to work for [company name]” on a 10-point Likert scale (1 = *do not agree at all*, 10 = *fully agree*). The expectation was that the answer to this question would depend on the participants’ overall motivation to do their best during the interview, and thus their motivational behavior expressed during the interview. We decided to use—from a psychometric perspective—a rather simplistic motivation assessment due to a lack of questionnaires on motivation to start working for a company (for potential alternatives, see “Discussion”). The self-reported motivation score was used to train the CBMM to objectively determine the participant’s motivation during the interview.

### Apparatus

The experiment was conducted on an HP Pavillion—17-ab455nd (Palo Alto, California, United States of America), using a custom-made online program in JavaScript that ran in a web browser. The video footage was collected using a Logitech (Lausanne, Switzerland) BRIO webcam at 1080p with 60 frames per second, using the Logitech Capture software. The experiment was conducted in a secluded, well-lit testing room at the university.

### Video analysis

Several facial markers were extracted from the videos of participants using a combination of algorithms integrated into OpenFace 2.1.0^53^. OpenFace allowed to extract a total of 17 action units (see Supplementary Table 1). Emotions were primarily computed with the use of EMFACS^54^ by combining AUs. The time traces of each action unit and emotion measurement were compressed to several singular valued properties (Fig. 1c), including the average activity of raw and high pass filtered data (i.e., subtracting a low-pass filter, removing a slowly changing baseline trend), the trend of activity (i.e., the slope of a linear fit to the time trace), the rate of emotion occurrences (i.e., activity peaks crossing a threshold of 1 SD above the median activity), and the average duration, area, and peak amplitude across all peak activities during the video.

### Recruiter ratings

To assess to what degree other people can determine the motivation of participants, we invited a total of 6 recruiters (M = 29.2, SD = 4.7, range = 23–36; 5 females; recruitment experience: M = 2.2 years, SD = 1.8, range = a few months to 4 years) to watch a total of 104 soundless interview videos of the participants. Recruiters did not watch all videos due to time and fatigue constraints. The recruiters read the motivation question that we also asked the candidates and then they rated each participant on how motivated they thought that the participant was to work for the company (same scale as candidate question). These judgments were used to train the RBMM.

### Modelling of motivation

To determine objectively how motivated participants were to apply for the company, we created a linear Lasso regression model with the action unit and emotion features as predictors (independent variables) and the candidate-ratings or recruiter-ratings of motivation as the predicted factor (dependent variable). Note that we had no priors to rely on as no literature exists that describes which action unit would be predictive of motivation. As the predictor set contained a large set of features, we applied dimension reduction and repeated (bootstrap-like) cross-validation to prevent under- and over-fitting (see Supplementary Figure S1b, for modelling steps). Dimension reduction was accomplished by selecting 30% of the features that correlated best with the motivation scores. Next, features with high variance inflation factors (> 10) were removed and replaced by a new feature in a stepwise, one-by-one manner, with a recalculation of VIFs after each feature replacement. We further removed or suppressed weights of less relevant features through Lasso regularization. To find a well-fitted model, we implemented a repeated cross-validation procedure with a novel training and test dataset division within each of the 1000 iterations per Lasso regularization setting. We created a total of 1000 times 20 Lasso regularization variations from an alpha (i.e., lambda) of log − 1 (no regularization; beta weights from an ordinal squares regression) to log 3 (extreme regularization; all weights suppressed to zero) and we inspected the root mean square errors (RMSE) as a function of the alpha parameter (Supplementary Figure S1c,e) to determine the degree of regularization that results in stable weights (Supplementary Figure S1d,f) and thus a well-fitted model. To simplify the model as a final step, we removed features with a significance value of above 0.75 in a stepwise fashion. To evaluate the final, full-fit model, we calculated the Spearman correlations between candidate-rated, recruiter-rated, and model-rated motivation scores. We applied signal detection theory^55^ to calculate hit, false alarm, and confusion rates across thresholds (i.e., between 0 and 100% percentile motivation scores).

## Supplementary Information

Supplementary Information.

## Data Availability

The data that support the findings of this study are available from Neurolytics BV but restrictions apply to the availability of these data, which were used under license for the current study, and so are not publicly available. Data are however available from the authors upon reasonable request and with permission of Neurolytics BV at marnixnaber@gmail.com.

## Code availability

The code that is used to generate the results of this study are available from Neurolytics BV but restrictions apply to the availability of this code, which is developed in collaboration with, and is part of the intellectual property of Neurolytics BV, and so is not publicly available. Enquiries can be sent to marnixnaber@gmail.com.

## Data transparency

The data used in the current study were collected during a larger experiment in which participants performed several aspects of an online assessment. The assessment consisted of, in addition to the interview examined in the current study, passively watching a company video, filling out questionnaires, and the performance of mental arithmetic tasks. The data collected in these other segments have yet to be analyzed and are thus not yet published nor submitted anywhere. We are however planning on starting analyses on these segments in the future with the aim of publication.
