# Supplementary material for: Objective and bias-free measures of candidate motivation during job applications
Source: Sci Rep. 2021 Nov 9;11:21254. doi: 10.1038/s41598-021-00659-y (PMC8578383; doi:10.1038/s41598-021-00659-y)
Supplement: Supplementary file 1 — Supplementary Information. [file 41598_2021_659_MOESM1_ESM.pdf]

**Appendix**

**Supplementary Table 1.** *Overview of action units and emotions.*

| Action unit / emotion | Corresponding action unit number |
|---|---|
| Inner brow raiser | 1 |
| Outer brow raiser | 2 |
| Brow lowerer | 4 |
| Upper lid raiser | 5 |
| Cheeck raiser | 6 |
| Lid tightner | 7 |
| Nosewrinkler | 9 |
| Upper lid raiser | 10 |
| Lip corner puller | 12 |
| Dimpler | 14 |
| Lip corner depresser | 15 |
| Chin raiser | 17 |
| Lip stretcher | 20 |
| Lip tightner | 23 |
| Lips part | 25 |
| Jaw drop | 26 |
| Blink | 45 |
| Happiness | 6, 12 |
| Sadness | 1, 4, 15 |
| Surprise | 1, 2, 5, 26 |
| Fear | 1, 2, 4, 5, 7, 20, 26 |

| | |
|---|---|
| Anger | 4, 5, 7, 23 |
| Disgust | 9, 15 |
| Contempt | 12[1], 14 |
| Interest | 1, 2, 5, 7, r20[2], r26 |
| Confusion[4] | 4, 7, 15, 17, 23 |
| Valence 1[5] | r1, r4, r5, 6, r10, r17, 12, 25, r45 |
| Valence 2[5] | 6, 12 |
| Arousal | Mean all AU with r45[6] |
| Blinks | 45[7] |

1. For contempt AU12 would only be activated on one side of the face. Dissociating left from right AU12 activations was, however, not possible due to technical limitations.

2. The letter r in front of an AU number indicates reversion (i.e., a higher activity corresponds to weakener corresponding emotion).

3. Interest is only measured at time points when a threshold activation of ≥0.4 for AU1 or AU2 was met.

4. Confusion threshold activation was set at ≥0.4 for AU4 and was met.

5. Two versions of valence were computed. The first was based on a pattern developed by McDuff and colleagues[51], and a second by Sayette and colleagues[52]

6. Action unit 45 was reversed because closing the eyes indicates a state of relaxation rather than arousal.

7. Blink threshold activation was set at ≥1 for AU45.

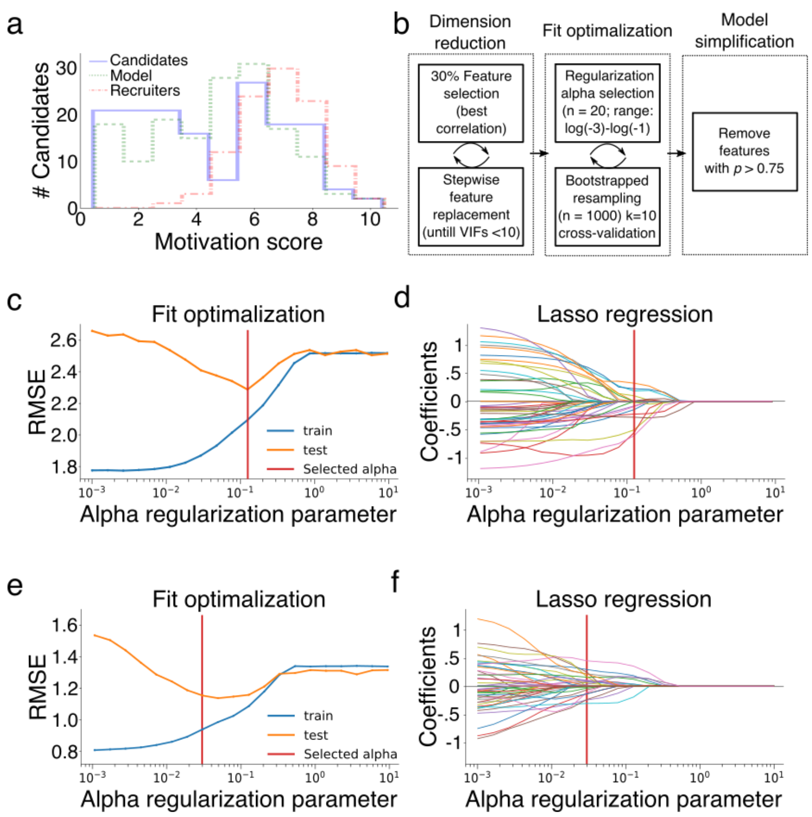


**Supplementary Figure S1. Motivation rating distributions, modelling steps, loss function and beta weights across regularization parameters. a**, Histogram of motivation scores of participants themselves (blue), objective model (green), and raters (red). **b**, Schematic drawing of modelling steps. **c**, Loss function showing the root mean square error (y-axis) for training set and test set as a function of the Lasso's alpha (lambda) regularization parameter (x-axis) for the model predicting candidates' self-reported motivation ratings. **d**, Coefficients (y-axis) of each feature as a function of alpha regularization parameter (x-axis). **e-f,** same as panel **c-d,** but now for the model predicting recruiters' motivation ratings. **d,f,** beta weights are strongly reduced at high alpha's to suppress features that have high collinearity with other features. The vertical red line indicates the selected alpha for the final, well-fitted model; this alpha selection results in a model for which the training and test RMSE were relatively similar, test errors were lowest, and weights of several features were decreased, all to prevent under- and over-fitting.
